# Indolent T‐cell‐rich small B‐cell hepatic lymphoma in a Golden Retriever

**DOI:** 10.1002/ccr3.1580

**Published:** 2018-06-06

**Authors:** Emily D. Rout, Kelly L. Hughes, Brendan O. Boostrom, Davis M. Seelig, Anne C. Avery, Paul R. Avery

**Affiliations:** ^1^ Department of Microbiology, Immunology and Pathology Colorado State University Fort Collins CO USA; ^2^ Department of Clinical Sciences Colorado State University Fort Collins CO USA; ^3^ Department of Veterinary Clinical Sciences University of Minnesota St. Paul MN USA

**Keywords:** canine, clonality analysis, flow cytometry, hematologic malignancy, immunophenotype

## Abstract

An 11‐year‐old female spayed Golden Retriever presented for an incidentally found liver mass. The hepatic mass and intra‐abdominal lymph nodes had a marked heterogeneous T‐cell population and far fewer numbers of small clonal B cells. This T‐cell‐rich small B‐cell lymphoma had a unique histological pattern and indolent clinical course.

## 1 INTRODUCTION

Lymphoma is one of the most common neoplasms in the dog, and many of the lymphoma subtypes seen in humans are also diagnosed in dogs.^1^ Veterinary medicine has adapted the World Health Organization (WHO) classification guidelines^2^ to diagnose lymphoma subtypes in animals,^3^, ^4^, ^5^, ^6^ but this classification system has been most thoroughly investigated in canine patients.^3^, ^7^, ^8^ The most common subtype in dogs is diffuse large B‐cell lymphoma.^3^, ^8^ Small B‐cell lymphomas and indolent lymphomas are recognized much less commonly in canine patients, and there have been few studies characterizing these rarer subtypes.^9^ One indolent lymphoma of T‐cell origin, T‐zone lymphoma, is being recognized more frequently now in dogs.^9^, ^10^, ^11^, ^12^, ^13^, ^14^ Indolent B‐cell lymphomas other than marginal zone lymphoma are still relatively uncommon,^1^, ^7^, ^8^, ^9^, ^14^ although this may partially be attributed to these subtypes being underdiagnosed due to their indolent behavior. Additionally, multicentric lymphoma, affecting multiple peripheral lymph nodes, is the most common presentation of canine lymphoma.^15^ Primary hepatic lymphoma is much less common and tends to have an aggressive clinical course.^16^, ^17^ This report describes a case of indolent small B‐cell lymphoma with marked T‐cell infiltrates, presenting in the liver and intra‐abdominal lymph nodes. This case contributes to our knowledge of indolent small B‐cell lymphoma in dogs and documents an unusual histological pattern with T‐cell‐rich infiltrates in this species.

## 2 CASE HISTORY/EXAMINATION

An 11‐year‐old female spayed Golden Retriever presented to the Colorado State University Veterinary Teaching Hospital (CSU‐VTH) for an incidentally found liver mass. Five days prior to presentation, she was evaluated at a veterinary clinic for a one‐day history of pollakiuria. At that visit, the patient was mildly painful on abdominal palpation. An abdominal ultrasound revealed a 5.2 × 5.0 cm round hypoechoic mass in the left lateral liver lobe, and a hypoechoic rounded structure possibly associated with the right middle liver lobe. A urinalysis collected by cystocentesis revealed two to three struvite crystals per high powered field and mild proteinuria, with no white blood cells or bacteria visualized. Empirical treatment with amoxicillin led to rapid resolution of her urinary signs. A complete blood count (CBC) and serum biochemistry profile were unremarkable. The patient had a history of bilateral elbow osteoarthritis and cranial cruciate ligament injury in both stifles and was enrolled in an ongoing CSU‐VTH clinical trial using mesenchymal stem cell therapy for dogs with osteoarthritis. Current medications included amoxicillin, and tramadol, amantadine, Metacam, fish oil, Dasuquin, and Cosequin for osteoarthritis.

On physical examination at the CSU‐VTH, the patient was bright and alert with a normal appetite and energy level. She was obese with a body condition score of 8/9. She had a weight bearing lameness of her left forelimb, localized to the elbow. No abnormalities were appreciated on abdominal palpation and peripheral lymph nodes palpated normally. An abdominal ultrasound at the CSU‐VTH confirmed the rounded, hypoechoic, heterogeneous, well‐vascularized hepatic mass on the left lateral aspect, measuring 4.5 × 5.7 cm. Thoracic radiographs were unremarkable.

## 3 DIFFERENTIAL DIAGNOSIS, INVESTIGATIONS, AND TREATMENT

**Figure 1:**
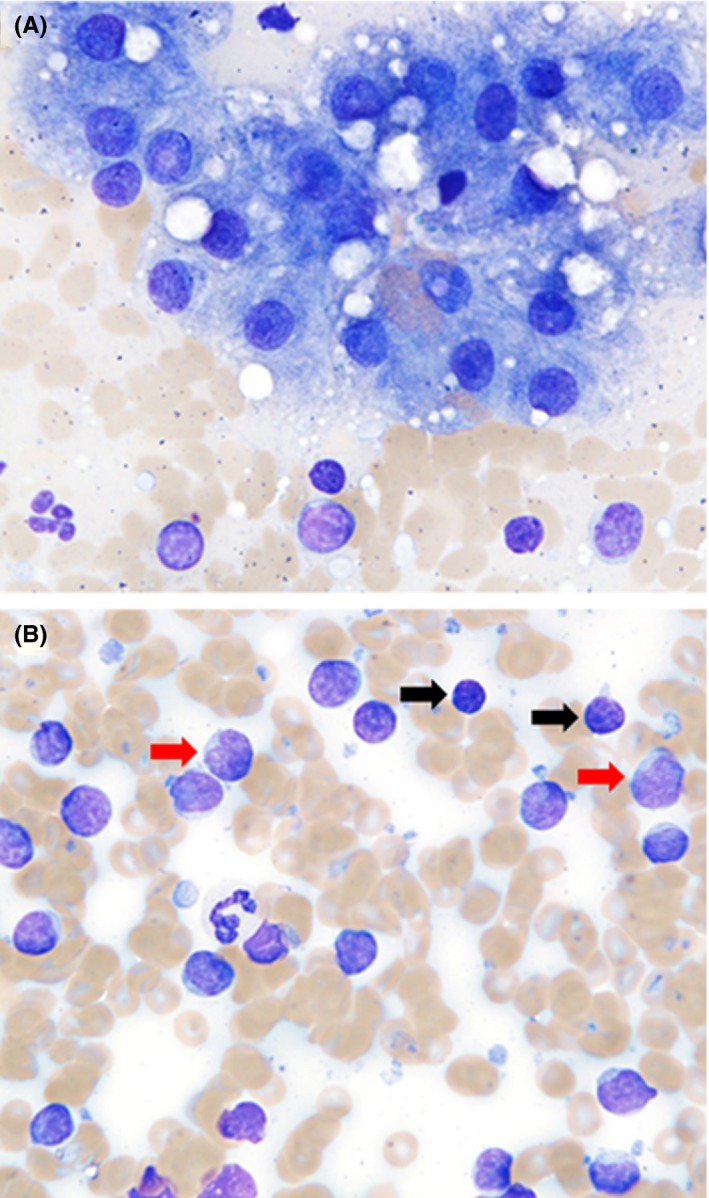
Cytologic pattern from a hepatic mass from a dog. Wright‐Giemsa, ×50 objective. Direct smears from a fine needle aspirate from a hepatic mass in a dog. There are clusters of well‐differentiated hepatocytes with mild vacuolar change (A), and increased lymphocytes in the background (B). The lymphoid population is heterogeneous, with small well‐differentiated lymphocytes (indicated by black arrows) and intermediate‐sized lymphocytes (indicated by red arrows)

Ultrasound‐guided fine needle aspiration of the hepatic mass was performed using a 22 gauge needle. Cytology revealed large numbers of lymphocytes which were predominantly intermediate‐sized, with smaller numbers of small lymphocytes (Figure 1). Intermediate lymphocytes were 12‐15 μm in diameter with a round to slightly indented nucleus, coarse chromatin, rarely one small faint nucleolus, and slightly expanded pale blue cytoplasm, which rarely contained few small azurophilic granules. Small lymphocytes were 8‐12 μm in diameter, with a small round nucleus, condensed chromatin, no apparent nucleoli, and scant amounts of basophilic cytoplasm. There was a pink amorphous background with abundant cytoplasmic fragments (lymphoglandular bodies), numerous bare nuclei from ruptured cells, and moderate blood contamination. There were rare small clusters of hepatocytes with mild cytoplasmic rarefaction, suggestive of hydropic change, and vacuolization. Fine needle aspiration of the adjacent hepatic parenchyma was evaluated cytologically and revealed small clusters of well‐differentiated hepatocytes with mild rarefaction and a large cluster of vacuolated biliary epithelium. An expanded lymphocyte population was not appreciated in this sample. The cytology of the liver mass was most consistent with lymphoma, although the presentation of a solitary hepatic nodule and mixed nature of the lymphocytes were unusual. Marked lymphocytic inflammation was considered much less likely.

**Figure 2:**
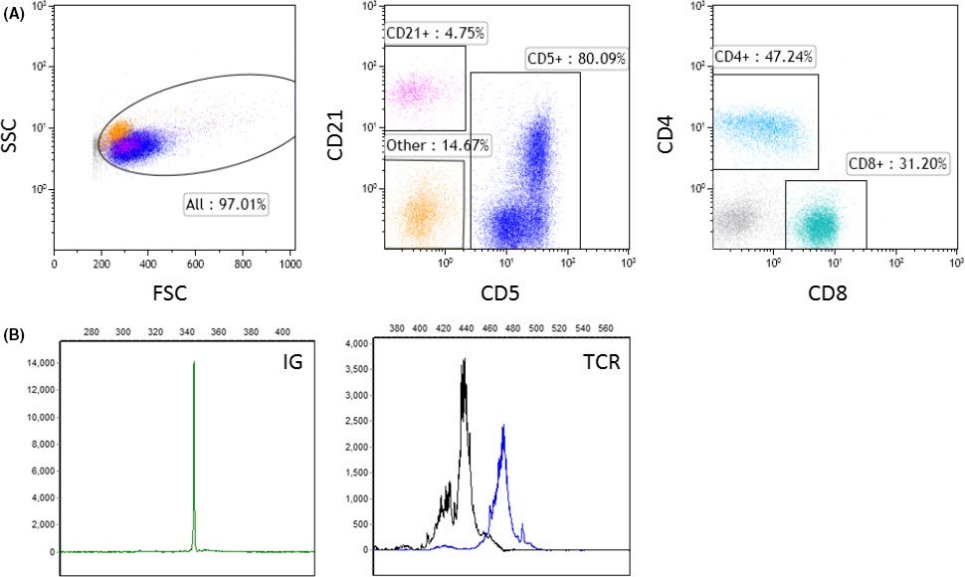
Flow cytometric immunophenotyping (A) and PCR for antigen receptor rearrangements (PARR) (B) results from a hepatic mass in a dog. (A) The size plot (left panel) with forward scatter (FSC) on the horizontal axis and side scatter (SSC) on the vertical axis shows the majority of cells in the population are small‐sized. Fluorescence dot plots show there is a predominance of CD5+ T cells (blue; middle panel), consisting of mixed CD4+ and CD8+ T cells (right panel). All CD5+ T cells expressed CD3 as well (not shown). There is a small population of CD21+ B cells (pink; middle panel) and a population of leukocytes (orange; middle panel) which express CD45 (pan‐leukocyte marker) and class II MHC (not shown). These cells do not stain with propidium iodide, indicating they are viable (not shown). (B) Immunoglobulin heavy chain (left panel) and T‐cell receptor (right panel) gene amplification of DNA from the hepatic mass. Results demonstrate a clonal immunoglobulin rearrangement and polyclonal T‐cell receptor rearrangements

Flow cytometric immunophenotyping was performed on a fine needle aspirate from the hepatic mass (Figure 2A), using previously described methods.^10^ T‐cell immunophenotyping antibodies available in the dog include anti‐CD3, anti‐CD5, anti‐CD4, and anti‐CD8. B‐cell antibodies for CD19 and CD20 are not available in the dog, but anti‐CD21 reliably detects B cells when used in combination with T‐cell antibodies^18^ and has emerged as an antibody commonly used for detecting B cells in most immunophenotyping laboratories.^10^, ^18^, ^19^, ^20^ By flow cytometry, there was a large expansion of lymphocytes, composed predominantly of mixed CD4+ T cells and CD8+ T cells, which were small in size. Approximately one‐third of CD3+ CD5+ T cells expressed low levels of CD21, which has been described in T‐cell subsets in other species.^21^, ^22^ There was a minor population of small CD21+ CD5− B cells, accounting for approximately 5% of the total cell population. Approximately 15% of the population consisted of small leukocytes expressing only CD45 (pan‐leukocyte marker) and class II MHC. Differentials for this phenotype include neoplastic lymphocytes or monocytes, early hematopoietic progenitor cells, natural killer cells, or plasma cells, although the light scatter properties are not consistent with monocytes or plasma cells. Neither extramedullary hematopoiesis nor significant plasma cell population were appreciated on cytology or subsequent histology. There were scant numbers of monocytes and neutrophils, which were attributed to a small amount of blood contamination. Given the heterogeneous expansion of small lymphocytes and the lack of lineage markers on the atypical population, a neoplastic lineage could not be identified and clonality testing was pursued using a PCR‐based assay^23^, ^24^ similar to the method used in human medicine.^25^ Clonality testing by PCR for antigen receptor rearrangements (PARR) revealed a clonal immunoglobulin rearrangement and polyclonal T‐cell receptor rearrangements (Figure 2B). The assay was performed in duplicate, revealing the same clonal immunoglobulin rearrangement.

**Figure 3:**
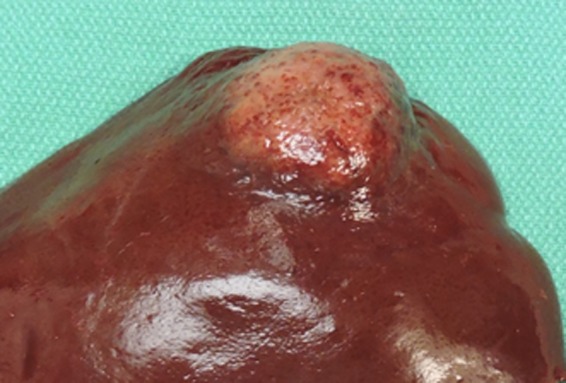
Gross image from a hepatic mass in a dog. The hepatic lobe is focally expanded by a discrete, soft, pale tan to white nodule, measuring 6 cm in diameter

One month after detection of the hepatic mass, the patient returned for abdominal exploratory surgery and liver lobectomy. A preoperative abdominal ultrasound found a second discrete mass in the left lateral liver lobe, which was hypoechoic and heterogeneous, measuring 3.1 × 4.9 cm. The previously described hepatic mass was unchanged. A CBC and serum biochemistry profile were unremarkable. The left lateral liver lobe containing the two discrete masses was surgically excised (Figure 3). Additionally, a 1 mm pale focus on the left medial liver lobe and the caudate process of the caudate liver lobe were biopsied and submitted for histopathology. A firm, enlarged portal lymph node and a prominent splenic lymph node were identified, and the lymph nodes were excised and submitted for histopathology. The patient recovered well from surgery and was discharged.

**Figure 4:**
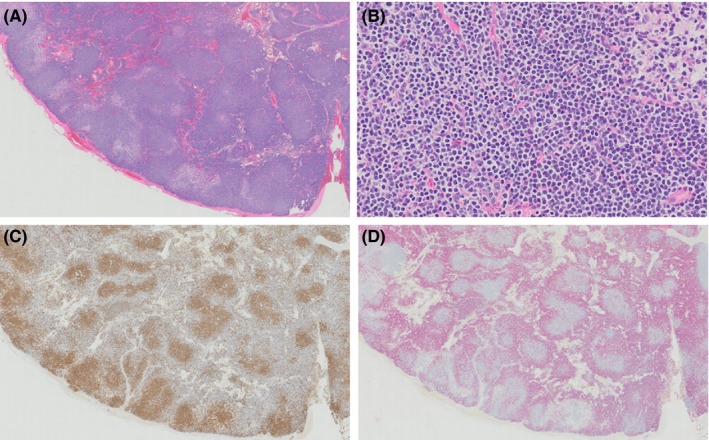
Histological and immunohistochemical findings of an intra‐abdominal lymph node in a dog with indolent B‐cell lymphoma. H&E, ×2x objective (A); H&E, ×20x objective (B); immunolabeling with anti‐CD3, 3,3′‐diaminobenzidine tetrahydrochloride chromogen, ×2x objective (C); immunolabeling with anti‐PAX5, fast Red chromogen, ×2x objective (D). Throughout the lymph node, there is an abnormal follicular pattern composed of central lighter‐staining populations of small to intermediate‐sized lymphocytes surrounded by darker bands of small lymphocytes (A). The cells in the central lighter areas (left, (B)) are small to intermediate‐sized (nuclei 1‐1.5 ×  size of a red blood cell) with condensed to slightly open chromatin and discrete cytoplasm and show immunoreactivity for CD3 (C). Cells surrounding these areas are slightly smaller with more condensed chromatin (right, (B)) and show immunoreactivity for PAX5 (D)

Histology of both lymph nodes revealed an expanded cortex with a nodular pattern (Figure 4). Follicular structures were present but abnormal, having a central lighter‐staining zone, surrounded by a dark‐staining zone occasionally compressed against vasculature and connective tissue. The lighter‐staining zone was not typical of a germinal center; the cells were monomorphic rather than a mixture of centrocytes and centroblasts and lacked polarity. Lymphocytes in this area were small to rarely intermediate‐sized (nuclei 1‐1.5× the size of a red blood cell) with condensed to occasionally more open chromatin, and scant to moderate amounts of homogeneous, eosinophilic, often discrete cytoplasm. The darker zone contained bands of a monomorphic population of small lymphocytes with nuclei 1‐1.25× the size of a red blood cell, densely clumped chromatin, indistinct nucleoli, and scant cytoplasm. There was clear space surrounding abnormal follicular structures, which was interpreted as edema. There were 0‐1 mitotic figures per high‐power field (×400). There was no evidence of capsular invasion. The medullary sinuses were dilated and congested and had multifocal areas of edema with numerous hemosiderophages.

**Figure 5:**
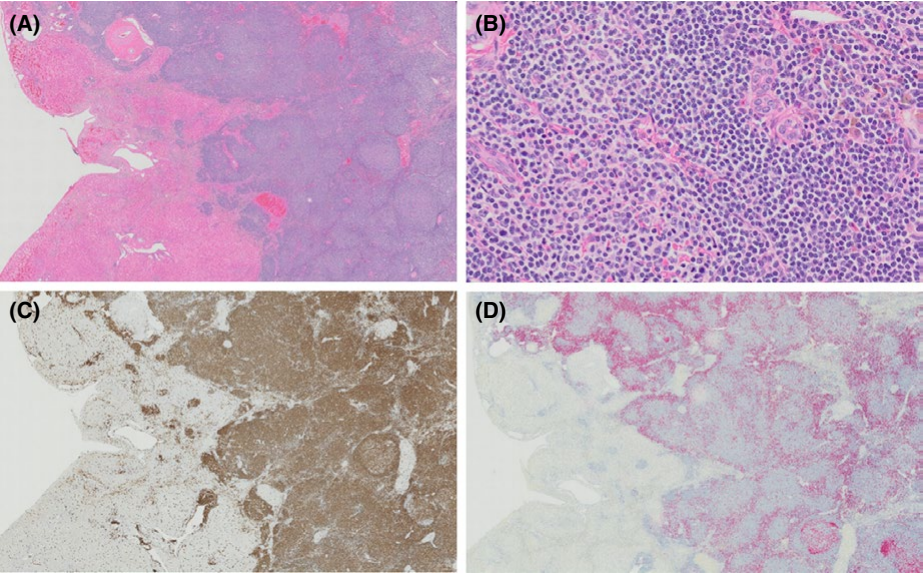
Histological and immunohistochemical findings from a hepatic mass in a dog with indolent B‐cell lymphoma. H&E, ×2x objective (A); H&E, ×20x objective (B); immunolabeling with anti‐CD3, 3,3′‐diaminobenzidine tetrahydrochloride chromogen, ×2x objective (C); immunolabeling with anti‐PAX5, fast Red chromogen, ×2x objective (D). The hepatic parenchyma is expanded by a nonencapsulated mass (A) composed of lymphocytes, arranged in an abnormal follicular pattern, similar to that described in the lymph node. Lymphocyte morphology is identical to the lymph node with small to intermediate‐sized cells with more discrete cytoplasm located centrally (left, (B)), and smaller cells surrounding these areas (right, (B)). The central areas making up the majority of the lymphocyte population are immunoreactive for CD3, including lymphocyte aggregates surrounding vasculature in the adjacent liver parenchyma (C). The smaller cells in the darker areas are immunoreactive for PAX5 (D) and represent a minority of the mass

Histology of the hepatic mass revealed replacement of the normal liver parenchyma by an expansile, nonencapsulated, fairly well‐demarcated mass (Figure 5). The mass was composed of lymphocytes, arranged in nodular structures with an atypical follicular pattern, similar to that described in the lymph node. Lymphocyte morphology was identical to that described in the lymph node, and there were 0‐2 mitotic figures per high‐power field (×400). There was mild to moderate, multifocal to coalescing hydropic degeneration in the surrounding hepatocytes. The liver capsule was irregular, and there were multifocal nodules of swollen hepatocytes demarcated by mild interstitial fibrosis (nodular regeneration). Portal areas frequently had mild oval cell hyperplasia and mild lymphohistiocytic infiltrates with histiocytes with cytoplasmic granular pigment.

Immunohistochemistry revealed the marked atypia of the follicular structures. In both the lymph node (Figure 4) and the liver (Figure 5), the central lymphocytes in the lighter‐staining zones had strong diffuse cytoplasmic staining for CD3 (monoclonal mouse anti‐human CD3, LN10 clone; Leica Biosystems Newcastle Ltd., Newcastle Upon Tyne, UK). The surrounding small lymphocytes in the dark‐staining zones had strong diffuse nuclear staining for PAX5 (monoclonal mouse anti‐human Pax5, DAK‐Pax5 clone; Dako North America Inc., Carpinteria, CA, USA). Immunohistochemistry for CD10 (monoclonal mouse anti‐human CD10, 56C6 clone; Leica Biosystems Newcastle Ltd.) was performed on the lymph node section at Purdue University. CD10 is considered a germinal center marker and is usually expressed in follicular lymphoma in humans.^2^, ^26^, ^27^ The few remaining normal lymphoid follicles contained lymphocytes with immunoreactivity for CD10 centrally, while all lymphocytes in the atypical follicular structures and interfollicular areas were negative.

The patient was diagnosed with a low‐grade small B‐cell lymphoma, with T‐cell‐rich infiltrates. An indolent lymphoma was considered most likely given the incidental identification of the hepatic mass, the low mitotic rate, and the silent clinical course of the disease.^9^ Therefore, no adjuvant treatment was initiated.

## 4 OUTCOME AND FOLLOW‐UP

At both 4 months and 1 year following surgical excision of the hepatic masses and lymph nodes, the patient was restaged with abdominal ultrasound, CBC, serum biochemistry profile, and urinalysis, which revealed no recurrence of lymphoma. There were no hepatic masses detected by ultrasound, and the lymph nodes were of normal size and echotexture. She had a grade II (low‐grade) cutaneous mast cell tumor completely excised from her neck 10 months after initial presentation. Twenty months after initial presentation, the patient presented with a four‐day history of epistaxis, and CBC revealed a severe thrombocytopenia (9000 platelets/μL; RI 200 000‐500 000/μL) with elevated mean platelet volume (17.6 fL; RI 7.5‐14.6 fL) and moderate normocytic, mildly hypochromic nonregenerative anemia (HCT 28%; RI 40%‐55%; CHCM 32 g/dL; RI 33‐36 g/dL). No abnormalities were detected on serum biochemistry profile, and there was a mild hyperlactemia (1.6 mmol/L; RI 0.20‐1.44 mmol/L) on the venous blood gas. Thoracic radiographs were unremarkable. Abdominal ultrasound revealed multiple well‐defined, round, hyperechoic, and hypoechoic heterogeneous masses in the liver, measuring up to 2.8 cm in diameter. The hepatic masses were not aspirated due to the severe thrombocytopenia. Cytology from a bone marrow aspirate revealed marked megakaryocytic hyperplasia, most consistent with ongoing loss, consumption, or destruction of platelets, and mild erythroid hyperplasia, possibly an early response to the anemia. Lymphocytes in the bone marrow were small and mature and accounted for 2.2% of total nucleated cells, which is considered normal. A serum ELISA test did not detect antibodies to *Ehrlichia*,* Anaplasma* or *Borrelia burgdorferi* organisms. A coagulation panel revealed normal PT and aPTT clotting times. Fibrinogen was elevated at 683 mg/dL (RI 123‐210 mg/dL),which was most likely attributed to inflammation. A low reticulocyte hemoglobin content (CHr) (22.1 mG/dL; RI 22.3‐27.9 mG/dL) also supported an inflammatory process.^28^ Immune‐mediated thrombocytopenia was the presumptive diagnosis, and immunosuppressive glucocorticoid therapy was initiated. The patient continued to decline and, given the ultrasound findings, the owners elected humane euthanasia the following day. On necropsy and histopathology, the liver masses were consistent with regenerative nodules with hepatocellular vacuolar degeneration. There was no evidence of lymphoma grossly or microscopically, indicating the patient was still in complete remission.

## 5 DISCUSSION

This report describes a case of indolent small B‐cell lymphoma with marked T‐cell infiltrates, forming solitary masses in the liver, and affecting intra‐abdominal lymph nodes. The histological pattern of this tumor does not fit clearly into the WHO classification system,^2^ which has been adapted for use in canine lymphoma.^3^, ^7^, ^8^ A number of diagnoses were considered, including T‐cell‐rich B‐cell lymphoma and lymphoma subtypes involving small B cells.

T‐cell‐rich large B‐cell lymphoma (TCRLBL) was considered given the marked T‐cell infiltrate in this case. In TCRLBL, the clonal B‐cell population can account for 10% or less of the total cell population and at least 50% of the total cell population is composed of T cells.^29^ However, the morphology of the B cells and the clinical course in this case were not consistent with human TCRLBL. In humans, TCRLBL is a subtype of diffuse large B‐cell lymphoma, with an aggressive clinical course and poor outcome.^30^ TCRLBL is rare in dogs and appears to have a variable clinical course, although there are few reports in the literature.^7^, ^14^, ^31^, ^32^ In one case report of a hepatic TCRLBL in a dog, the patient was less than a year old, the neoplastic B cells were large in size, and there was poor response to chemotherapy with the patient dying 28 days after the start of chemotherapy.^32^ However, Flood‐Knapik et al reported a case of TCRLBL surviving 27.4 months without chemotherapy.^14^ TCRLBL is the most common lymphoma subtype reported in horses, where further studies are needed to determine the clinical behavior.^5^ In cats, Hodgkin’s‐like lymphoma, which can also have a heterogeneous lymphoid infiltrate with rarer neoplastic B cells like TCRLBL, is reported and appears to have a prolonged clinical course.^33^ Therefore, TCRLBL may have a more variable clinical course in veterinary species compared to humans. However, we did not think this case was consistent with TCRLBL histologically. The neoplastic B cells in TCRLBL are large, and there is often a histiocytic component, and neither of these features were present in this case.

Two other B‐cell lymphoma subtypes that can have a rich T‐cell infiltrate in people are extranodal marginal zone lymphoma and follicular lymphoma.^34^, ^35^ Marginal zone lymphoma and follicular lymphoma are diagnosed in dogs as well,^9^, ^13^, ^14^, ^36^, ^37^ although significant T‐cell infiltration appears to have only been described in canine nodal marginal zone lymphoma.^13^ In human patients, both cutaneous and noncutaneous extranodal marginal zone lymphoma can have a predominance of T cells and arise within a background of chronic inflammation due to infection or autoimmune disease.^38^, ^39^, ^40^ In this case, there was no cutaneous involvement, the B‐cell population did not have the classic single prominent nucleolus or expanded cytoplasm typical of marginal zone cells,^7^, ^9^ and the degree of T‐cell infiltration appeared more pronounced than that described for MZL in humans and dogs. Marginal zone lymphoma can have an inverted follicular pattern, but in those cases, the follicle is described as having a central dark‐staining zone surrounded by a light‐staining outer zone,^41^ which is opposite of the atypical follicular pattern identified in this case. Follicular lymphoma often has an intermixed infiltrate of T cells and wide variation in follicular shape and pattern. However, the center of the follicular structures should contain a disorganized mixture of B cells, including centrocytes and centroblasts,^42^ with interfollicular areas composed of residual T cells of the paracortex.^7^ In this case, the follicular pattern was due to a central population of T cells with surrounding B cells, and few germinal centers were evident. CD10 is one of the markers often used in the diagnostic workup of follicular lymphomas in humans.^42^ In follicular lymphoma, CD10 expression is often strong within follicular structures, but may be decreased or absent in the interfollicular neoplastic B cells.^43^ CD10 immunohistochemistry was pursued in this case because of the follicular pattern and the few remaining normal follicles were positive for CD10, but the vast majority of B cells in this neoplasm were negative for CD10.

Other differentials for mature small B‐cell neoplasms with an abnormal T‐cell component were also considered, including chronic lymphocytic leukemia/small lymphocytic lymphoma (CLL/SLL),^44^ lymphoplasmacytic lymphoma, and mantle cell lymphoma.^2^, ^45^ CLL/SLL can have an indolent disease course, but generally has a diffuse histological pattern and lymphocytosis, which was not present in this case. Bone marrow was not available for histological examination in this case, but lymphocytes were within normal limits in the bone marrow cytologically. The histological features and clinical features described in lymphoplasmacytic lymphoma and mantle cell lymphoma were inconsistent with this case.^46^

Finally, due to the heterogeneous lymphocyte population, autoimmune lymphoproliferative syndrome (ALPS) was also considered. ALPS is a disorder described in people in which lymphocyte homeostasis is disrupted, resulting in nonmalignant lymphoproliferation in the lymph node, liver, and/or spleen, including expansions of CD4−/CD8− T cells.^47^, ^48^ Autoimmune conditions frequently manifest, including autoimmune hemolytic anemia and thrombocytopenia, and patients have an increased risk of lymphoma. This condition occurs at an early age in people and has an underlying genetic component, associated with pathogenic variants in Fas pathway genes.^47^, ^49^ This entity has not been described in dogs, and several features in this case are discordant with ALPS. This patient was older at presentation, the B‐cell population was clonal, and the T‐cell infiltrate consisted of mixed CD4+ and CD8+ T cells, with no CD4−/CD8− T cells detected by flow cytometry. This patient ultimately developed severe thrombocytopenia, which may have been immune‐mediated, but this occurred 20 months after diagnosis with no evidence of lymphoproliferation during the interim. Immune‐mediated thrombocytopenia is a complication of lymphoproliferative diseases of B‐ and T‐cell origin in people, particularly in CLL/SLL.^50^, ^51^ Unfortunately, we were unable to confirm that the thrombocytopenia in this case was immune‐mediated, and there was no evidence of lymphoma at the time of thrombocytopenia, although immune‐mediated thrombocytopenia can occur after complete remission of lymphoma in human patients.^52^

The clonal immunoglobulin gene rearrangement in this case is consistent with B‐cell lymphoma. Pseudoclonal PCR results are possible when a sample contains a minor population of B cells, but the clonal product was reproducible in duplicate reactions, supporting a diagnosis of B‐cell malignancy.^53^ The cytology was consistent with lymphoma, but unusual given the heterogeneous morphology of the lymphocytes. The histology and immunohistochemistry were suspicious for T‐cell lymphoma, given the number of T cells present, but flow cytometry was useful for determining the T‐cell population was actually a heterogeneous mixture of CD4+ and CD8+ T cells. Clonality testing by PARR was ultimately necessary to identify the clonal B cells. By flow cytometry, this clonal B‐cell population may either be represented by the small numbers of CD21+ B cells, and/or the population expressing CD45 and class II MHC.

This case report describes a small B‐cell lymphoma with a marked T‐cell infiltrate in a dog, with an indolent clinical course, which was apparently cured by surgical excision of the hepatic masses and affected lymph nodes. The anatomic distribution, histological pattern, and clinical course of this case appear unusual for both veterinary and human medicine. Although several lymphoma subtypes were considered for further classification, the histological pattern and clinical features of this case did not appear to fit well with a current subtype. This case also highlights the utility of additional diagnostics, including flow cytometry and PARR, for identifying the reactive and clonal components of a lymphoid neoplasm and obtaining an accurate diagnosis.

## CONFLICT OF INTEREST

None declared.

## AUTHORSHIP

EDR: pathologist, collected and analyzed data, performed the literature review, and wrote the manuscript; KLH: pathologist, analyzed data, performed the literature review, and wrote parts of the manuscript; BOB: oncologist, involved in the patient’s care; DMS: pathologist, analyzed data; ACA: performed flow cytometry and clonality testing, analyzed data; PRA: pathologist, involved in data analysis and manuscript preparation. All authors involved in the revision and final approval of the manuscript.
